# Emotional engagements predict and enhance social cognition in young chimpanzees

**DOI:** 10.1111/desc.12145

**Published:** 2014-01-11

**Authors:** Kim A Bard, Roger Bakeman, Sarah T Boysen, David A Leavens

**Affiliations:** 1 Centre for Comparative and Evolutionary Psychology, University of PortsmouthUK; 2 Department of Psychology, Georgia State UniversityUSA; 3 Department of Psychology, The Ohio State UniversityUSA; 4 School of Psychology, University of SussexUK

## Abstract

Social cognition in infancy is evident in coordinated triadic engagements, that is, infants attending jointly with social partners and objects. Current evolutionary theories of primate social cognition tend to highlight species differences in cognition based on human-unique cooperative motives. We consider a developmental model in which engagement experiences produce differential outcomes. We conducted a 10-year-long study in which two groups of laboratory-raised chimpanzee infants were given quantifiably different engagement experiences. Joint attention, cooperativeness, affect, and different levels of cognition were measured in 5- to 12-month-old chimpanzees, and compared to outcomes derived from a normative human database. We found that joint attention skills significantly improved across development for all infants, but by 12 months, the humans significantly surpassed the chimpanzees. We found that cooperativeness was stable in the humans, but by 12 months, the chimpanzee group given enriched engagement experiences significantly surpassed the humans. Past engagement experiences and concurrent affect were significant unique predictors of both joint attention and cooperativeness in 5- to 12-month-old chimpanzees. When engagement experiences and concurrent affect were statistically controlled, joint attention and cooperation were not associated. We explain differential social cognition outcomes in terms of the significant influences of previous engagement experiences and affect, in addition to cognition. Our study highlights developmental processes that underpin the emergence of social cognition in support of evolutionary continuity.

## Research highlights

- Two types of social cognition, joint attention and cooperation, develop in chimpanzees in the first year of life.
- Previous engagement experiences and concurrent affect are major proximate mechanisms for social cognition outcomes in young chimpanzees.
- Cognition plays an important role for joint attention, especially at younger ages, but not for cooperation.
- Cooperation and other triadic skills are not proximate mechanisms for joint attention skills, above and beyond engagement experiences and affect.
- Important developmental processes apply to social cognition in chimpanzees, supporting evolutionary continuity among hominoids.

## Introduction

Joint attention (JA), infants' triadic ability to engage with social partners about objects or events in the world (Bakeman & Adamson, 1984), emerges between 9 and 12 months of age in humans, and marks the onset of shared intentionality (Tomasello, Carpenter, Call, Behne & Moll, 2005). Current formulations of the Shared Intentionality model assume that social cognition is built upon the cognitive construct of the psychological understanding of other minds, and further claim that joint attention relies on uniquely human cooperative motives, and that the capacity to engage jointly with another is a biological adaptation unique to humans (Hamann, Warneken, Greenberg & Tomasello, 2011; Tomasello & Moll, 2010). Here we propose the Lived Experiences model of social cognition to be applied to nonhuman, as well as human, primates. This model is based on the ontogeny of human social cognition as empirically documented by developmental and comparative psychologists (e.g. Adamson & Bakeman, 1985; Tomasello *et al*., 2005) and focuses on the proximate processes by which infants develop engagement with persons and engagement with objects, and coordinate these engagements in the phenotypic expressions of cooperation and joint attention. The Lived Experiences model predicts different phenotypic outcomes in primate social cognition (Bard & Leavens, 2009; Jablonka & Lamb, 2007; Kellogg & Kellogg, 1933; Leavens & Bard, 2011), based on social learning mechanisms that are simple or complex (Meltzoff, Kuhl, Movellan & Sejnowski, 2009; Moore & Corkum, 1994; Papousek, 2007; Rumbaugh, King, Beran, Washburn & Gould, 2007; Roseberry, Richie, Hirsh-Pasek, Golinkoff & Shipley, 2011) and may depend on affect (Adamson & Bakeman, 1985; Deák, Triesch, Krasno, de Barbaro & Robledo, 2013; Panksepp, 2011; Syal & Finlay, 2011). With the application of this developmental model, we explain how nonhuman primate social cognition develops through interactional experiences with social partners, living within particular eco-cultural settings (Bakeman, Adamson, Konner & Barr, 1990; Hewlett, Fouts, Boyette & Hewlett, 2011; Keller, 2007; Racine & Carpendale, 2007), by highlighting the constructs of affect and previous engagement experiences, in addition to cognition (Adamson & Bakeman, 1985; Trevarthen & Aitken, 2001). This model is empirically derived from prototypic human development and here is applied for the first time to chimpanzee development of joint attention and cooperation.

With the Lived Experiences model, we embrace developmental principles for social cognition outcomes that are achieved with rapid learning, without explicit teaching, based on actual experiences, and that both contribute to and result from neural connectivity networks (Bard, 1994; Panksepp, 2011; Syal & Finlay, 2011; Striano & Reid, 2009). Of course, cognition depends on supportive neural structures, but we know that neural structures develop in interaction with environmental influences (Fox, Levitt & Nelson, 2010). We suggest that the learning processes involved in the early development of social cognition among hominoids (apes and humans) are based on largely shared evolutionary mechanisms (e.g. Bard, 2009; Gardner, 2005; Jablonka & Lamb, 2007).

The Lived Experiences model predicts different social cognition outcomes for chimpanzees that live in different ecological and social settings. Cross-species studies of social cognition have not typically controlled for postnatal experiences (e.g. age at testing, past learning experiences, or quality of rearing/engagement experiences; Call & Tomasello, 2008; Hamann *et al*., 2011; Herrmann, Call, Hernandez-Lloreda, Hare & Tomasello, 2007; Povinelli & Eddy, 1996; Tomasello & Carpenter, 2005). When the social cognition of young (family-raised) humans is compared with adult (laboratory-raised) chimpanzees, differences cannot be logically attributed solely to species, as species is confounded with duration (and quality) of postnatal experiences (Bard & Leavens, 2009; Kellogg & Kellogg, 1933; Leavens & Bard, 2011; Leavens, Bard & Hopkins, 2010; Leavens, Hopkins & Bard, 2005). This problem might be trivial if, in apes, postnatal experiences did not impact social cognition. However, ape-language and cross-fostering projects provide irrefutable evidence of the significant impact of lived experiences on cognitive outcomes in apes; e.g. some chimpanzees learn symbol systems (Fouts, 1997; Gardner, Gardner & Van Cantford, 1989; Premack, 1976; Rumbaugh, 1977; Savage-Rumbaugh, Murphy, Sevcik, Brakke, Williams & Rumbaugh, 1993), and enculturated adult apes perform significantly better than laboratory-raised apes in tests of both social cognition and physical cognition (Bania, Harris, Kinsley & Boysen, 2009; Furlong, Boose & Boysen, 2008; Lyn, Russell & Hopkins, 2010; Russell, Lyn, Schaeffer & Hopkins, 2011). Postnatal experiences in apes have been linked with differential outcomes that rely on joint attention, such as pointing (Leavens & Bard, 2011; Lyn *et al*., 2010; Leavens *et al*., 2010; Gardner *et al*., 1989), imitation, and emulation (Bjorklund, 2006; Furlong *et al*., 2008; Gardner, 2005; Rumbaugh, Washburn, King, Beran, Gould & Savage-Rumbaugh, 2008; Russell *et al*., 2011; Savage-Rumbaugh *et al*., 1993). Common processes have been proposed to explain developmental changes, e.g. pointing emerges in human infants and in some apes as a solution within the Referential Problem Space (Leavens *et al*., 2005), the environmental circumstance in which an organism is reliant on others to act on the world for them (e.g. babies in a crib, older apes in a cage).

Conceptualizations of early social cognition in humans include a range of skills involving joint attention (JA) and cooperation. Initial reports of JA skills focused on overt behaviors such as infants' abilities to follow the gaze, head orientation, and pointing of adults, and infants' coordinated joint engagements with social partners and objects (Bakeman & Adamson, 1984; Adamson & Bakeman, 1985; Corkum & Moore, 1995). More recently, research on JA skills has shifted to focus on underlying psychological motivations, such as infants' communicative intentions (e.g. declarative, imperative, informative; Leavens & Bard, 2011), or infants' motivations for engagement (Tomasello *et al*., 2005; Tomasello & Moll, 2010; Hamann *et al*., 2011). To be useful in comparative research, however, constructs must include overt behaviors, i.e. operational definitions. Skill sets based on theoretical constructs alone, without corresponding behavioral anchors, are particularly problematic (Butterfill, 2012; Leavens & Bard, 2011; Racine & Carpendale, 2007). For example, a mental state is often discussed as something tangible that can be shared with others. It is only when such constructs are defined with behavioral markers, observed and measured, that they can be determined to be manifest in human or nonhuman primates (Deák *et al*., 2013; Leavens, Hopkins & Bard, 2008).

Our focus in this paper is the emergence and development of JA and cooperation in the first year of life, with the goal to elucidate those processes that support the young infant's developing social cognition. We view these two skills globally as requiring coordinated joint engagement, in other words, as coordinating infants' object-related activity with that of their social partner, especially so as to benefit from demonstrations, and from both verbal and gestural guidance (Adamson & Bakeman, 1985; Bakeman & Adamson, 1984). We use a behavioral measure of cooperation, defined as the ‘give and take’ activity commonly observed in pre- and non-verbal infants (e.g. Adamson & Bakeman, 1985; Tomasello & Moll, 2010). Whereas the topic of JA is typically the object (e.g. how to act on the object), the topic of cooperation is more about the social activity (the social exchange, the activity of passing back and forth between social partners; e.g. Bakeman *et al*., 1990; Bayley, 1969). Developmental researchers, from 1960 through the 1980s, routinely found that cooperativeness was an important index of the infant's involvement with test taking, which, in turn, influenced cognitive performances (Meisels, Cross & Plunkett, 1987).

Since we take a developmental perspective, we hypothesize that both JA and cooperation are grounded in foundational domains, including previous experience, affect, and cognitively important dyadic skills (Adamson & Bakeman, 1985; Tomasello *et al*., 2005; Trevarthen & Aitken, 2001). We suggest that affect is critically important, as it indicates the quality of the infant's engagement with the test-taking task (e.g. Bayley, 1969; Meisels *et al*., 1987). For both human infants and chimpanzee infants, testing is routinely stopped if the infants do not settle, or if they become distressed (i.e. if they are overly fearful or predominantly unhappy), and therefore, affect can be considered an index of basic readiness to engage with a test. Our study also addresses the empirical relation between cooperation and JA, evaluating whether they are mutually interdependent as claimed by the Shared Intentionality model (e.g. Tomasello & Moll, 2010). To further elucidate infants' triadic skill sets, we assess triadic skills involved in physical cognition (infant-object-object). Thus, we can evaluate whether social cognition and physical cognition develop independently (an implicit assumption in much of comparative psychology; e.g. Call & Tomasello, 2008; Herrmann *et al*., 2007).

Here we evaluate the proximate processes underlying joint attention and cooperation by studying changes across development, and comparing outcomes in two groups of chimpanzees that were given systematically different postnatal engagement experiences. Previous studies of ape social cognition have not documented the features of pre-experimental experiences that might impact differential social cognitive outcomes, with the exception of language-like skills (Lyn *et al*., 2010; Russell *et al*., 2011). Here the chimpanzees were subjected to a controlled manipulation of early experiences during the first year of life and were not given any training in artificial symbol systems. Virtually all other studies in this domain are post-hoc comparisons of apes that have had different rearing experiences, and are therefore merely correlative; hence, ours is a uniquely powerful prospective study.

The soundness of applying the Lived Experiences model is evaluated by a comparison of social cognition outcomes in these two groups of chimpanzee infants, with normative profiles of human infants (Bayley, 1969), in which we control for the amount of postnatal experience (i.e. groups are age-matched). Our overall strategy is to specify a model of cognitive development grounded in normative human developmental profiles and apply that model to a large sample of chimpanzee infants. This Lived Experiences model of chimpanzee social cognition will be rejected if we find (1) no within-species differences, that is, if the two chimpanzee groups do not differ, demonstrating that chimpanzees are insensitive to our experimental rearing history manipulation; (2) large cross-species differences, that is, phenotypic expressions are not comparable in chimpanzees and humans; or (3) that social cognition in chimpanzees is based on different developmental processes or developmental patterns from those of humans.

## Material and methods

### Participants

#### The chimpanzee samples

Forty-nine chimpanzee infants, born from 1987 to 1995 at the Yerkes National Primate Research Center (henceforth: Yerkes Center), Emory University, were the subjects of this study. The policy at the Yerkes Center during this period was for all chimpanzee infants to be raised by their biological mothers, whenever possible. However, when females did not exhibit sufficient maternal competence to ensure infant survival (Bard, 1994), their infants were raised in the Great Ape Nursery, with either Standard Care (ST: *n *=* *32), or Responsive Care (RC: *n *=* *17) nursery regimes, where their interactional experiences with humans differed (Bard, 1996).

**Figure 1:**
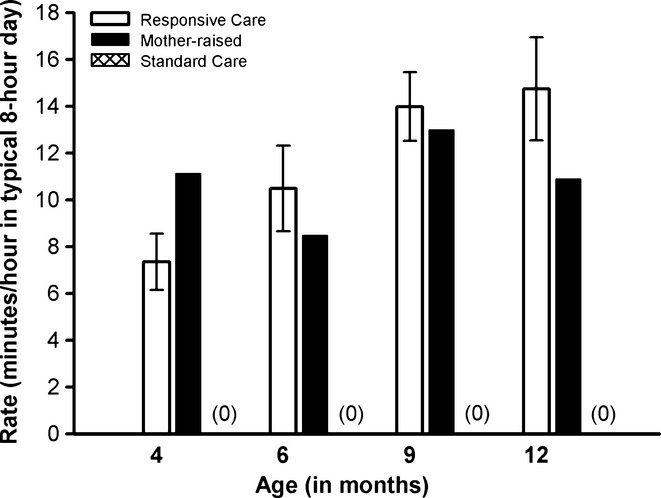
Infants' experiences of engagement with caregivers' encouragement of species-typical skills. The columns are the rates (minutes per hour based on an 8-hour day) during which infant chimpanzees experienced their caregiver support, encouragement, or nurturing of species-typical development (i.e. a summation of caregiver time spent nurturing motor skills, nurturing social skills, nurturing communicative skills, and nurturing general autonomy). The open columns are the average rates for caregivers of responsive care chimpanzees (at 4 months, n = 13, at 6 months, n = 16, at 9 months, n = 17, at 12 months, n = 16), with 99% confidence intervals. Solid columns are the rates for chimpanzee mothers (based on a total of 8 hours of observation), and the 0s indicate the average rates for caregivers of standard care chimpanzees. At 6 and 12 months of age, RC infants experienced these nurturing interactions significantly more often than mother-raised infants (ps <.01). At 9 months, RC infants experienced nurturing as much as mother-raised infants (p > .05), and at 4 months, mother-raised infants experienced more nurturing than RC infants (p < .01).

The engagement histories of the chimpanzees are quantifiable in terms of the amount, quality, and particular types of human–chimpanzee infant interactions across the two nursery rearing protocols (see Supplementary Information [SI], for more detail). The ratio of infants to caregivers was much lower in Responsive Care (RC: 1 or 2 infants to 1 caregiver) than Standard Care (ST: ∽13 infants to 1 caregiver), and RC infants spent much more time with caregivers (300 minutes of caregiver–infant contact per weekday) than ST infants (60 minutes of caregiver–infant contact per day). The most important difference was in the types of interactions (Figure1). RC infants, but not ST infants, experienced species-typical communicative interactions with caregivers who were trained to interact in chimpanzee-appropriate ways (RC: 20 hrs/week; ST: absent). For example, RC researchers spent between 7 and 15 minutes per hour nurturing the development of species-typical chimpanzee skills, which compares favorably to 8.5 to 13 minutes/hour spent by mother chimpanzees at the Yerkes Center (Figure1), and reports from wild mother–infant interaction (van Lawick-Goodall, 1968). ST caregivers did not spend any time encouraging species-typical chimpanzee skills, because they had a considerable number of other duties, and the ST philosophy was that infants would learn chimpanzee species-typical behavior from peers. At 4, 6, 9, and 12 months of age, RC infants experienced these nurturing interactions significantly more often than ST infants (all *p*s <.01). Note that the engagement experiences of both nursery- and mother-raised chimpanzee infants at the Yerkes Center were distinctly different from those of wild chimpanzees (Boesch, 2012; van Lawick-Goodall, 1968), of chimpanzees raised in other biomedical laboratories (Bard, Brent, Lester, Worobey & Suomi, 2011; Kalcher, Franz, Crailsheim & Preuschoft, 2008), of sanctuary chimpanzees (Ferdowsian, Durham, Kimwele, Kranendonk, Otali, Akugizibwe, Mulcahy, Ajarova & Johnson, 2011), and of home-raised chimpanzees (Fouts, 1997; Hayes, 1951; Kellogg & Kellogg, 1933; Temerlin, 1976). Note that here the chimpanzee infants did not learn any artificial symbol systems.

#### The human sample

The human infants, whose data are used here, constituted the standardization sample used in the development of the Bayley Scales of Infant Development (BSID; Bayley, 1969). The number of infants tested at each age varied (5 months, *n *=* *42; 6 months, *n *=* *57; 8 months, *n *=* *48; 10 months, *n *=* *49; 12 months, *n *=* *52). Bayley (1969) stated that they were selected from diverse socioeconomic backgrounds and tested with the BSID from 1958 to 1960. The infants were predominantly white (85%), from urban (69%) rather than rural settings, and lived in two-parent-family homes in the USA (100%). Data reported here were computed from Tables 12 and 17 of the manual (Bayley, 1969). Note that the rearing experiences of these human infants were distinctly different from the experiences of human infants from non-US cultures (e.g. Bakeman *et al*., 1990; Gaskins, 2006; Greenfield, Keller, Fuligni & Maynard, 2003; Hewlett *et al*., 2011; Keller, 2007) and of human infants from orphanages (Kaler & Freeman, 1994; Leiden Conference on the Development and Care of Children without Permanent Parents, 2012; Rutter, Sonuga-Barke, Beckett, Castle, Kreppner, Kumsta, Scholtz, Stevens, Bell & Gunnar, 2010), with differential consequences for social, cognitive, and emotional outcomes (Fox *et al*., 2010; Nelson, Zeanah, Fox, Marshall, Smyke & Guthrie, 2007).

### Procedures

#### Bayley Scales of Infant Development

The Bayley Scales of Infant Development (BSID; Bayley, 1969) is the most widely used test for assessing cognitive development of pre- and non-verbal infants. The BSID consisted of 102 items for testing 5- to 12-month-old human infants, and was administered to all participants using published procedures (administration and scoring of relevant items are described briefly in Tables S1–S4, abstracted from Bayley, 1969). Procedures included administration in a quiet room by a trained examiner who was at ease with infants, beginning at a basal level (with approximately 10 items that the infant would be expected to pass), and stopping after approximately 10 items were failed (recorded on the MDI scoring form). Tests lasted between 20 and 30 minutes.

##### Mental Development Index (MDI)

The BSID results are typically reported simply as a Mental Development Index (MDI) score, comparable to an IQ score for older children and adults. MDI scores are derived from tabled values (pp. 110–115 in Bayley, 1969) computed from raw scores (total number of BSID items passed added to the basal value) as a function of infant age (MDI score for the average typically developing human at each age was standardized to 100).

##### Infant behavior record (IBR)

Immediately after each test, the examiner completed the IBR form (Bayley, 1969), rating the infant's behavior along several dimensions according to standard criteria. IBR data were available at all eight ages for the chimpanzees. Human IBR scores at 5, 6, 8, 10, and 12 months were computed from the data available in Table 17 of the BSID manual (Bayley, 1969).

#### Administration and reliability

Chimpanzee infants were tested by three different examiners, each trained by the same examiner who was an expert tester with human subjects. All examiners were blind to the hypotheses of the current paper. An initial examiner (VB) tested only ST infants (57 tests). The second examiner (KG) tested both ST infants (144 tests) and RC infants (57 tests). A third examiner (JA) tested only RC infants (145 tests). Fifteen of 16 multivariate ANOVAs revealed no significant or systematic differences between examiners (eight analyses between VB and KG with testing of ST infants, all *p*s *ns*; seven analyses between KG and JA with testing of RC infants, *p*s *ns*) in the scoring of cooperativeness, fearfulness, and positive emotional tone. Very good reliability on the MDI portion of the BSID was found in comparisons between the expert human examiner and the primary chimpanzee examiner on raw scores (percent agreement 94%). There were no differences in both raw scores and MDI scores between examiners (paired *t*-tests, *p*s *ns*), and very high correlations (.97,.88, *p*s <.001). These two types of reliability estimates demonstrated that the chimpanzee examiners were: (1) consistent and unbiased in the scoring of IBR items; and (2) not different from human examiners in the scoring of the BSID items (see SI for more information).

### Data reduction

In addition to the MDI, we derived seven additional scores for subsequent analysis, four from the BSID, and three from the IBR. As described below, we categorized individual BSID items by the type of skill each required and then assigned items to the four BSID-derived variables, depending on the skill category. Their score was the sum of the items passed, computed for the chimpanzees from the BSID score sheets, and for the humans by counting the number of items passed by the typical infant, from 5 months to 12 months of age, as reported by Bayley (1969; and adapted in Tables S1–S4). Classifying items of the BSID in this way, according to their essential skills, is unique to our study. To the best of our knowledge, neither human nor chimpanzee BSID data have been reported in this way previously.

#### Joint attention

The *joint attention* score was based on 30 BSID items (see Table S1). In order to pass joint attention (JA) items, the infant was required to coordinate attention to the examiner (and/or her actions) with attention to the test object(s). The specific form of the coordinated joint engagement ranged from imitating the examiner's actions with a test object, to following the examiner's gestures and/or verbal request concerning test object activity or the required actions with test objects. We are confident that the JA items are valid measures of the skill, as they included both the coordination of attention (demonstrated by gaze alternation between the examiner's face and the test object: see SI, Figures S7 and S8), and the coordination of infant activity with that of the social partner (demonstrated by passing the items; Striano & Reid, 2009; Wieder & Greenspan, 2003).

#### Cooperativeness

*Cooperativeness* was assessed with Item 4 from the IBR. Scores of 1 to 3 indicated that the infant most often refused to cooperate, scores of 4 to 6 indicated that the infant accepted the give-and-take of objects with the examiner, and scores of 7 to 9 indicated that the infant actively took part in the give-and-take exchange with enjoyment.

#### Affect: positive emotional tone and fearfulness

Two scores that indexed affect were derived from the IBR. What we call *positive emotional tone* was assessed with Item 7, General Emotional Tone from the IBR (Figure S1). A score of 3 indicated that the infant was unhappy at times during the test, a score of 5 indicated general contentment, and a score of 7 indicated that the infant was generally happy. *Fearfulness* was assessed with Item 5 from the IBR (Figure S2). A score of 3 indicated that the infant was vigilant most often during the test, a score of 5 indicated that the infant showed moderate amounts or moderate durations of fear, and a score of 7 indicated that the infant was frequently and/or intensely fearful.

#### Cognitive dyadic BSID scores

Two scores indexed dyadic skills. First, *dyadic social skills* were based on eight BSID items (see Table S3). These social items assess basic expression of emotion, basic social responses, and basic communicative expressions, reflective of the infant as a social being (e.g. reacting to a social partner or reacting with social responses). Second, simple *object manipulation skills* were based on 25 BSID items. At younger ages, these items included looking at, reaching toward, and grasping objects; by 12 months, they included finely detailed manipulation, such as exploring the details of a bell, and purposive manipulation of single objects, such as turning the pages of a picture book, and moving a bell to generate ringing sounds.

#### Cognitive triadic scores

The social triadic skills, joint attention and cooperativeness, have already been discussed. Cognitive non-social triadic skills (infant-object-object), which we call complex *object-object manipulation*, were assessed with 13 BSID items (see Table S4). These items required the infant to act on an object in relation to, or in a functional relation with, a second object. Examples of these triadic items include lifting a cup to reveal a hidden toy and placing a round block into the circular hole on a form board.

#### General cognition

Finally, we retained the MDI score as an index of general cognition, summarizing the overall performance of an infant, i.e. their general developmental status. We reasoned that MDI scores might not be equivalent to the sum of the extracted items, although this working hypothesis is subject to empirical verification in our analyses.

### Data analyses

#### Multilevel trajectory analyses

Our design called for testing chimpanzees eight times, at monthly intervals between 5 and 12 months of age, but some tests were missed (Bard & Gardner, 1996). For ST chimpanzees, the number of months for which scores were available averaged 5.75 (*SD* = 2.22, range 2–8), whereas for RC chimpanzees, the average was 7.96 (*SD* = 1.22, range 7–8). Accordingly, we estimated trajectory parameters (slopes, intercepts, confidence intervals) for this multilevel model (monthly scores for the eight scales described in the previous section nested within chimpanzee, with chimpanzees in either the ST or RC group) with MPLUS, a program that, *inter alia*, can estimate parameters for multilevel structural equation models when the number and timing of repeated observations varies (Heck & Thomas, 2009; Muthén, 1997; Raudenbush & Bryk, 2002).

For each of the eight scales, we proceeded as follows. To determine whether ST and RC groups differed, first we fitted a model that constrained slopes (change in score per month) for the two groups to be identical. This level of the analysis tells us whether the intercepts for ST and RC groups differ, and whether the slopes for the eight scales differ significantly from zero (indicating significant developmental change). A second model allowed slopes to differ, in which developmental trajectories in the two chimpanzee groups were compared. A 99% confidence interval was constructed on the resulting regression lines.

Unlike the longitudinal chimpanzee data, the human data derived from Bayley (1969) were cross-sectional and with varying sample sizes (42 to 57). To compare the human data with the chimpanzee data, we note where the human scores fall relative to the confidence intervals (CI) for the chimpanzee data. Specifically, we regard the human sample as significantly different from the chimpanzee groups when the human data fell outside of the 99% CI surrounding the regression lines for the chimpanzee groups.

#### Hierarchic multiple regression analyses

One goal of this study was to investigate the role of different processes in the development of social cognition. In order to assess the unique contributions of these developmental processes (i.e. group membership, affect, cognitive dyadic skills, cognitive triadic skills, and overall cognitive ability), with respect to predicting joint attention and predicting cooperativeness in the chimpanzees, we employed hierarchic multiple regression (Cohen & Cohen, 1983). In hierarchic multiple regression, the order in which variables are entered is specified in advance. In particular, we did not use computer-determined, data-driven methods that determine order by proportions of variance accounted for (*R*^*2*^). Here, the hierarchical order was based on prototypic human development. Our *a priori* hypothesis was that previous engagement experiences (i.e. group membership: ST vs. RC) and concurrent affect (positive emotional tone and fearfulness during the test) were predictors of joint attention and cooperation outcomes, and, thus, these variables were entered as the first, second, and third steps, respectively. By entering these variables first, their effects were controlled in subsequent steps investigating the independent effects of cognitive variables on outcomes. The remaining order was predetermined, entering cognitive dyadic skills, cognitive triadic skills, and general cognition in that order. For the dyadic and triadic skills, social skills were added before the object skills because social skills typically occur earlier in development (Bakeman & Adamson, 1984). The hierarchical nature of the analysis meant that the unique contribution of each variable was determined, above and beyond the variables already in the model (i.e. the reported statistic was change in the proportion of variance accounted for, *ΔR*^*2*^, at each step). Effects on outcome of variables entered later in the model statistically controlled for variables entered earlier.

To determine the effect of engagement history, the outcome variable (either joint attention or cooperativeness) was regressed first on a coded variable for group (step 1). A significant *ΔR*^*2*^ at this first step would mean that being raised in RC or ST, in itself, was predictive of social cognition outcomes. Positive emotional tone was added in step 2 and fearfulness, the other affect scale, was added in step 3. Affect scores were added before others because of affect's early occurrence in development (Adamson & Bakeman, 1985; Trevarthen & Aitken, 2001), and our theoretical position that affect is critically important in the development of primate social cognition. We predicted that previous engagement history (Group) and concurrent emotion (Affect) would be important predictors of both joint attention and cooperation outcomes, i.e. if so, then these first three steps would account for a large proportion of unique *R*^*2*^. The following analyses, thus, will test these theoretical predictions: The Lived Experiences model will be rejected if significant *ΔR*^*2*^ is not associated with previous experience and affect.

With previous experience and concurrent affect controlled, we entered a series of variables that collectively measured infant cognition. The cognitive dyadic skills were added; specifically, dyadic social skill and dyadic object manipulation skill were steps 4 and 5, respectively. A significant *ΔR*^*2*^ would indicate that the infant's dyadic cognitive skills, after controlling for engagement experiences and affect, were important in predicting joint attention skills or cooperation. The cognitive triadic social skill scales were added in step 6 (i.e. either cooperativeness when joint attention was the outcome or joint attention when cooperativeness was the outcome) and triadic object-object manipulation skills in step 7. A significant *ΔR*^*2*^ at these steps would indicate that infants' triadic skills were important in predicting JA or cooperation, after controlling for engagement experiences, affect, and dyadic skills. According to the Shared Intentionality model, cooperation underpins joint attention in humans, and therefore, this aspect of their theoretical model was explored here. This aspect of their model would be rejected for chimpanzees if a significant *ΔR*^*2*^ were not found at step 6. The final step added was general cognition, i.e. the MDI score. A significant *ΔR*^*2*^ at this final step would indicate that general intelligence, above and beyond all the other variables, uniquely predicted the social cognition outcome.

The design of the study called for the chimpanzees to be assessed eight times, at monthly intervals from 5 to 12 months. The goal of the hierarchic multiple regression analyses was to ascertain those processes that underlie the development of joint attention and those that underlie the development of cooperation. Developmental change in the foundational skills and in the joint attention and cooperation outcomes was expected. However, conducting hierarchic multiple regression analyses at each age was inadvisable, due to the relatively small sample size and the relatively large number of analyses that would result. If there was no developmental change, then variables could be averaged over the 8 months, and a single hierarchic multiple regression analysis could be conducted for each outcome. However, preliminary analyses suggested significant developmental change in many of the variables (Figures S1–S6). Accordingly, we conducted two hierarchic multiple regression analyses for each outcome, first using variables averaged across the first four months (i.e. from 5 to 8 months, inclusive), and second, using variables averaged across the last 4 months (i.e. from 9 to 12 months, inclusive). Thus, we could determine whether the underlying processes contributing to joint attention and those contributing to cooperation were different during the earlier and later developmental periods of the study.

## Results

### Joint attention success

**Figure 2:**
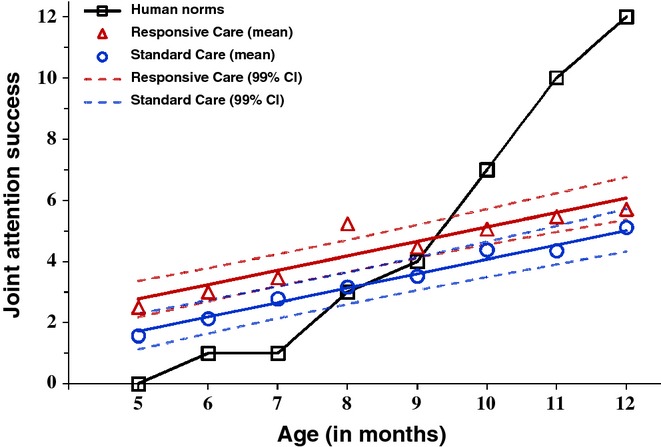
Joint Attention Developmental Trajectories. The average number of joint attention items passed (JA Success indicated by symbols), developmental trajectories (slope of change with age, shown in solid lines) and 99% confidence intervals (dashed lines) are shown for 28 *Standard Care* (in blue) and 17 *Responsive Care* (in red) chimpanzees, and for human norms (in black) tested from 5 months to 12 months of age.

Both groups of nursery chimpanzees were significantly more successful in joint attention (JA) than the human infants, at 5, 6, and 7 months of age (Figure2: *p*s <.01). The chimpanzee groups were successful in JA at an earlier age than the human infants: Chimpanzees were less than 5 months and humans were 6 months when they passed the first JA item. Human JA success was significantly lower than the Responsive Care (RC) chimpanzees at 8 and 9 months (*p*s <.01). Human infants were significantly more successful in JA than both chimpanzee groups from 10 through 12 months of age (*p*s <.01). The developmental trajectory of JA success increased significantly in all groups (*p*s <.001), but it was steeper for humans (*b *=* *1.71) than for the chimpanzee groups (ST *b *=* *0.51; RC *b *=* *0.51). JA success was significantly higher in RC chimpanzees, given enriched engagement experiences, than in Standard Care (ST) chimpanzees with essentially no overlap in 99% confidence intervals, and significantly different intercepts.

Two hierarchic multiple regressions were conducted to determine the unique contribution of foundational variables to Joint Attention (JA: Table1). In predicting JA at the younger age period (5–8 months), a significant *ΔR*^*2*^ of 19% was found for previous engagement experiences (i.e. group), and a significant *ΔR*^*2*^ of 13% also was found for Affect (fearfulness). In predicting JA at the older age period (9–12 months), a significant *ΔR*^*2*^ of 8% was found for previous experiences (i.e. group), and a significant unique *ΔR*^*2*^ of 24% was found for Affect (positive emotional tone). In terms of cognitive predictors, a significant *ΔR*^*2*^ was found for dyadic social skills at both age periods, 13% and 9%, respectively, above and beyond the variance accounted for by previous experience and affect. In predicting JA at the younger age, but not the older age, a significant *ΔR*^*2*^ of 6% was found for dyadic object manipulation skill, after controlling for group, affect, and dyadic social skills. Triadic skills (cooperativeness and complex object-object manipulation skills) did not make a significant unique contribution at either age period, after controlling for engagement history, affect, and dyadic skills. General cognition accounted for significant *ΔR*^*2*^ of 12%, only at the younger age period, after controlling for all other variables. The variables as a set accounted for 66% and 43% of joint attention variance at younger and older ages, respectively (59% and 31% when adjusted for the sample size and number of predictors). The full model was significant at both younger and older ages, *F*(8, 36) = 8.84, *p *<* *.001, and *F*(8, 38) = 3.62, *p *=* *.003, respectively.

**Table 1:** Predicting joint attention and cooperativeness in nursery-raised chimpanzees with hierarchic multiple regressions

Step	Predictor variable	*ΔR*^2^				*β*			
		Joint attention		Cooperativeness		Joint attention		Cooperativeness	
		5–8 mo	9–12 mo	5–8 mo	9–12 mo	5–8 mo	9–12 mo	5–8 mo	9–12 mo
1	Group (engagement history)	19**	8*	14*	21**	.43**	.29*	.38*	.46**
2	Positive emotional tone	0.2	24**	62**	33**	.05	.57**	.92**	.67**
3	Fearfulness	13**	1	2*	∽0	–.89**	.21	–.38*	.03
4	Dyadic social skill	13**	9*	0.3	2	.44**	.38*	.06	.17
5	Object manipulation skill	6*	∽0	4**	2	.42*	.02	.33**	.24
6	Triadic social skill	∽0	0.4	∽0	0.3	–.04	.09	–.02	.07
7	Object-object manipulation	3	0.6	∽0	2	.24	.10	.00	.17
8	General cognition	12**	0.5	0.1	0.1	.87**	.13	.11	–.05
	Total *R*^2^	**66**	**43**	**83**	**60**	**—**	**—**	**—**	**—**
	Adjusted *R*^2^	**59**	**31**	**79**	**52**	**—**	**—**	**—**	**—**

Note:*N *=* *45 at 5–8 months and *N *=* *47 at 9–12 months. Statistics for steps 1–8 are proportion of additional unique variance accounted for expressed as a percentage (*ΔR*^2^) and the regression coefficient (*β*) when each variable is added to the equation, from a hierarchic multiple regression with order of entry specified as shown. Total *R*^2^ and total *R*^2^ adjusted for number of variables and sample size are likewise expressed as percentages. Significant *ΔR*^2^ and β are indicated with bold;

* *p *<* *.05;

** *p *<* *.01.

### Cooperation

**Figure 3:**
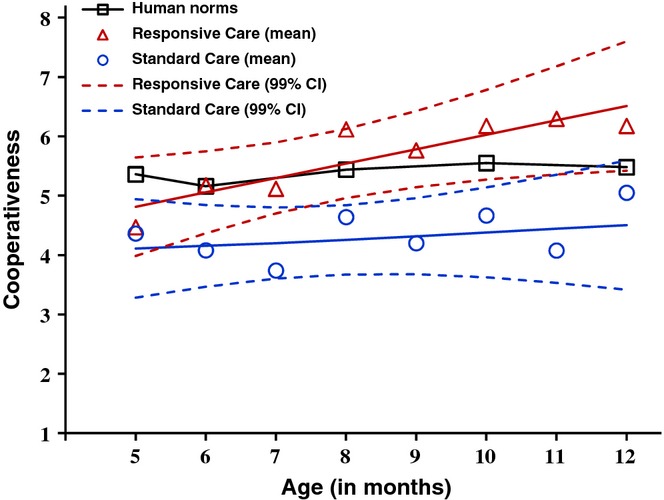
Cooperativeness Developmental Trajectories. Cooperativeness mean scores (symbols), developmental trajectory (solid lines) and 99% confidence intervals (dashed lines) are shown for 28 standard care chimpanzees (in blue), 17 responsive care chimpanzees (in red), and 42–57 human infants (in black) tested from 5 months to 12 months of age.

Cooperativeness scores for the human infants were within the 99% confidence interval of RC chimpanzees except at 12 months, when human scores fell just below the lower 99% boundary for RC, and just above the upper 99% boundary for ST chimpanzees (Figure3). Cooperativeness was significantly higher in RC than ST chimpanzees (significantly different intercepts, *p *<* *.001). In humans and ST chimpanzees, the developmental trajectory of cooperativeness was flat (*b *=* *0.02, *b *=* *0.06, respectively), but it increased in RC chimpanzees (*b *=* *0.24). The developmental trajectory in cooperativeness of RC chimpanzees was steeper than that of ST chimpanzees, approaching but not reaching statistical significance (*p *=* *.058).

The hierarchic multiple regression analyses showed that previous engagement experiences (group) accounted for significant *ΔR*^*2*^ of 14% and 21% at the younger and older ages, respectively (Table1). Positive emotional tone made unique contributions to cooperativeness at both developmental periods, accounting for significant *ΔR*^*2*^ of 62% and 33%, respectively, above and beyond the variance accounted for by group membership. At the younger age period, but not the older age period, fearfulness and cognitive dyadic object skills accounted for significant, but small, increases in *R*^2^ of 2% and 4%, respectively. Neither type of triadic skill nor general cognition contributed to cooperativeness, after controlling for engagement history, affect, and dyadic skills. The variables as a set accounted for 83% and 60% of cooperativeness variance (79% and 52%, when adjusted for the sample size and number of predictors), for the younger and older developmental periods, respectively. The full model was significant at both younger and older ages, *F*(8, 36) = 21.82, *p *<* *.001, and *F*(8, 38) = 7.16, *p *<* *.001, respectively.

### Joint attention–cooperation link

The hierarchic multiple regression analyses revealed that JA and cooperation were not empirically associated in the chimpanzees at 5–8 months or at 9–12 months, after statistically controlling for the effects of engagement history, affect, and dyadic skills. Although significant bivariate correlations between JA and cooperativeness occurred (*r *=* *.31 and.49, *p *=* *.04 and <.01, at the younger and older ages, respectively), the direct association disappeared when variables entered earlier in the regressions were taken into account. Both social cognition outcomes were related significantly to engagement experiences and to affect. In chimpanzee infants, the link between cooperation and joint attention success, at both age periods, was fully explained by previous engagement experiences, affect, and dyadic social skills.

There was not a strong association between the development of joint attention and cooperation in young human infants, since the developmental trajectories of JA and cooperativeness differed dramatically. For the human infants, their steeply rising joint attention trajectory (*b *=* *1.71) was not accompanied or matched by any developmental change in cooperativeness, which had a flat slope (*b *=* *0.02). For the human infants, flat developmental trajectories were found in affect (Figures S1 and S2) and general cognition (Figure S6). Rising slopes were found for three aspects of cognitive development: both types of dyadic skills (Figures S3 and S4), and non-social triadic skills (Figure S5). If JA and cooperation were interdependent across development, then the sharp increases in JA skills would be matched by similar developmental changes in cooperation, which was not found. It is possible, of course, that a direct link exists, but only in older children, or that only a minimal level of cooperation is required to support joint attention. However, note that by 12 months, the RC chimpanzees excelled in cooperation compared to the humans, but the RC chimpanzees did not excel in joint attention, suggesting that enhanced cooperation does not lead directly to enhanced JA.

## Discussion

Our results were strongly consistent with the Lived Experiences model: social cognition outcomes differed within chimpanzees based on their previous engagement experiences; there was substantial comparability in the social cognition outcomes of chimpanzee infants and human infants in the first year of life; social cognition outcomes in the chimpanzees showed significant developmental change from 5 to 12 months of age; and, most importantly, both concurrent emotional engagement and previous engagement experiences were foundational for social cognition outcomes in young chimpanzees. The Lived Experiences model, a human-based model of developmental processes leading to shared intentionality, was applied successfully to chimpanzees. Thus, our findings lend support to claims of evolutionary continuity in developmental processes that subserve social cognition (Bard, 2009; Boesch, 2012; Gardner, 2005; Gómez, 2005; Hopkins, Russell & Schaeffer, 2012; Jablonka & Lamb, 2007).

We found a spurt in joint attention success in the human infants from 10 months of age that was not matched by the chimpanzees, providing support for a quantitative difference between laboratory-raised chimpanzee infants and typically developing US infants. It is unclear whether this difference is attributable to: (a) the limits of the institutional settings of the chimpanzees; or (b) psychological limits in chimpanzees. Because joint attention success in these laboratory chimpanzee infants was systematically malleable in the presence of differential engagement experiences (see below), we suggest that the former possibility is more likely. Group differences in joint attention success cannot be attributed to length of postnatal experiences or different types of assessment, because all participants were given the same test, with the same durations of postnatal experience (i.e. at the same chronological ages).

We cannot confirm species differences in JA success, however, because the human and chimpanzee groups differed systematically in the quality of postnatal engagement experiences: the human infants were raised in dual-parent-family homes in the US (Bayley, 1969), and the chimpanzee infants were raised primarily in peer-group nurseries, under institutional conditions of a biomedical laboratory, which are known to impact negatively on cognition (Leavens & Bard, 2011; Lyn *et al*., 2010; Rumbaugh *et al*., 2008; Russell *et al*., 2011). Of particular importance, are the differences in attachment relations: The majority of US infants in the 1950s had a functioning attachment relationship (with 65% secure attachments), whereas the majority of the nursery-reared chimpanzees exhibited disorganized attachment (at levels similar to those of human infants raised in the worst of orphanages: van IJzendoorn, Bard, Bakermans-Kranenburg & Ivan, 2009). Ape infants and ape adults with non-institutional, enriched experiences exhibit significantly greater competencies (Bania *et al*., 2009; Fouts, 1997; Furlong *et al*., 2008; Menzel, Davenport & Rogers, 1970; Savage-Rumbaugh *et al*., 1993), suggesting that institutionalized rearing inhibits social cognition in apes, as it does in humans (Brune, Brune-Cohrs, McGrew & Preuschoft, 2006; Kalcher *et al*., 2008; Kaler & Freeman, 1994; Leavens & Bard, 2011; Fox *et al*., 2010; Nelson *et al*., 2007; Rutter *et al*., 2010). In other studies with engagement histories more closely matched than was possible here, human infants, even at 2.5 years of age, did not excel in social cognition relative to apes (Russell *et al*., 2011). The Lived Experiences model is useful in explaining this range of phenotypic variation in joint attention as a function of engagement history.

We found that cooperation in the human infants did not show improvement across early development. The give-and-take exchanges that defined cooperativeness here were shown to be typical for young human and chimpanzee infants; however, it should be acknowledged that cooperation can continue to develop into collaboration in children and human adults (e.g. Hamann *et al*., 2011), with some outcomes unparalleled in any other primate (Whiten & van Schaik, 2007). Ontogeny and years of post-toddler interactional experiences are crucially important for human infants to become collaborators and intentional beings (Racine & Carpendale, 2007; Tomasello & Moll, 2010). However, the results reported here strongly support the conclusion that postnatal experiences during early development are proximate mechanisms supporting the development of cooperation, intentionality, and intersubjectivity in apes, as well as in humans (Bakeman *et al*., 1990; Bard, 2012; Boesch, 2012; de Waal & Ferrari, 2010; Fouts, 1997; Hayes, 1951; Henrich, Heine & Norenzayan, 2010; Kellogg & Kellogg, 1933; Leavens *et al*., 2010; Lillard, 1998; Papousek, 2007; Trevarthen & Aitken, 2001; Watson, 1972).

An enriched engagement history, even within an otherwise austere institutional setting, significantly enhanced both joint attention and cooperation in the chimpanzee infants. Experiences of nurturing were presented here (Figure1) to illustrate one type of engagement experience that differed significantly for chimpanzee infants raised in a standard care (ST) nursery versus a responsive care (RC) nursery. There were two major routes through which engagement experience impacted social cognition, directly (the nursery group variable reflects the totality of their different engagement experiences) and indirectly, via changing infants' affective states during the test. Affect during the test was not only a significant factor in both social cognition outcomes, above and beyond group experiences, but also a very meaningful factor (i.e. accounting for large percentages of unique variance: 13% and 24% in joint attention, and 62% and 33% in cooperation across the two age periods). Affect, both positive emotional engagement and lack of fearfulness, were concurrent measures of the infants' emotional engagement with the test-taking task. Interestingly, it was previous experience that primarily predicted JA at the younger ages, and primarily positive emotional tone that predicted JA at the older ages. We conclude that a major aspect of the application of a Lived Experiences model to chimpanzee development is supported, because previous engagement experiences and concurrent emotional engagement were significant and unique variables in the development of social cognition for young chimpanzees.

We found that cognition was also an important predictor for joint attention. According to developmental principles, poor social cognition could occur due to infants' (1) poor social skills, since both joint attention and cooperation require a strong foundation of engagement with a social partner; (2) poor object manipulation skills, since joint attention assessed here required objects to be manipulated in particular ways; (3) poor ability to form triadic relations, since cooperation and joint attention were both defined by the triangle of infant-object-social partner; or (4) a general cognitive deficit. We considered these four different aspects of cognition separately in order to be more specific about their influences. The significant hierarchic multiple regression models for joint attention and cooperation included all these developmentally assessed cognitive skills, after controlling for engagement history and affect. At the younger age of 5–8 months, the sum of all the cognitive skills accounted for 34% of the variance in JA success (approximately equal to the combined effects of previous experience and concurrent affect), and at the older age of 9–12 months, the sum of all the cognitive skills accounted for 11% of the variance (much less than the 32% of the variance accounted for by previous experience and concurrent affect). These analyses extend our knowledge of how infants develop social cognition by highlighting dyadic, rather than triadic, skills as underpinning JA especially, but also Cooperation, to a lesser extent. Based on our findings, we suggest that the developmental processes that subserve the emergence of joint attention and cooperation are similar and conserved in hominoid evolution.

The primacy of engagement experiences and positive affect in predicting Cooperativeness scores at both periods contrasts with the range of variables predicting Joint Attention, suggesting that there may be important differences in the developmental processes underlying different types of social cognition, at least in chimpanzees. Moreover, the contributions of underlying processes change across developmental periods, which presents intriguing challenges for developmental research with humans as well. Studies exploring the influence of emotion and previous engagement experiences on developmental change in social cognition would illuminate important processes in human infants (Deák *et al*., 2013; Trevarthen & Aitken, 2001).

The theoretical construct of ‘motivation to share things with others psychologically’ is proposed by Tomasello *et al*. (2005, p. 689) to be a core species-unique concept of the Shared Intentionality model (Tomasello & Moll, 2010). Such theoretical constructs appear speculative, and are difficult to apply in comparative research when operational definitions are lacking (Deák *et al*., 2013). One aspect of this sharing construct, Cooperativeness, is indexed here by the amount and enjoyment of ‘give-and-take’ between infant and adult. Prototypic western human infants are proposed to show joy in sharing, for its own sake (e.g. Carpenter & Call, 2013; Herrmann *et al*., 2007), although similar behaviors have been observed in some apes as well (reviewed in Bard & Leavens, 2009). When matched for duration of postnatal experience, we found that the species did not differ in this measure of sharing, nor did they differ in positive emotional tone during testing (even though their engagement histories differed with a demonstrable impact on emotional expressions, such as social smiling; Bard *et al*., 2011). Emotional and social engagement histories are undoubtedly important variables of the ‘motivation for sharing’ construct, and our study has shown that this is empirically true for the ‘give-and-take’ type of sharing in chimpanzees, as measured here. We suggest that future research could explore the extent to which engagement history and concurrent emotion are linked with cooperative motivations in human infants. Thus, the Lived Experiences model is useful in operationalizing the theoretical construct of ‘motivation to share with others psychologically’, which is also important in support of future comparative research.

We suggest that joint attention and cooperation develop within particular eco-cultural settings for human and chimpanzee infants, and that variation in the phenotypic expressions of social cognition can be explained by social learning mechanisms involved in actual experience (Deák *et al*., 2013; Rumbaugh *et al*., 2008). The within-species variation in early development is illustrated by the significant differences found between the two groups of nursery-raised chimpanzees tested here (Bard, 2012; van IJzendoorn *et al*., 2009). The extraordinary capabilities for advanced physical and social cognition displayed by great apes that are raised in human-like artifactual and emotional landscapes (Lyn *et al*., 2010; Rumbaugh *et al*., 2008; Russell *et al*., 2011), combined with the significant group differences in both joint attention and cooperation among the institutionalized chimpanzees reported here, lead to the clear conclusion that great apes' socio-cognitive skills are highly influenced by as-yet-poorly-specified features of engagement experiences (e.g. interactions within their early rearing environments and emotional engagements), in addition to cognitive skills. It is important to emphasize that the immense range in cognitive outcomes reported for chimpanzees cannot be attributed to purely genetic factors (Boesch, 2012; Jablonka & Lamb, 2007; Leavens & Bard, 2011; Rumbaugh *et al*., 2008). In applying the Lived Experiences model, we advocate for the inclusion of the often-ignored variables of emotion, engagement, learning, early experience, and development into evolutionary models of primate social cognition.

## Author note

KAB designed and conducted the chimpanzee studies, and conceived the comparative analyses; KAB and DAL prepared the manuscript to which STB and RB made substantial contributions; RB, with KAB, conceived and conducted the analyses, and prepared the graphs; STB and KAB designed and supervised the eye gaze data collection.

## Supporting Information

Data S1 Emotional Engagement Enhanced and Predicts Joint Attention and Cooperation in Young Chimpanzees
