# Supplementary material for: Emotional engagements predict and enhance social cognition in young chimpanzees
Source: Dev Sci. 2014 Jan 11;17(5):682–96. doi: 10.1111/desc.12145 (PMC4116479; doi:10.1111/desc.12145)
Supplement: Data S1 — Emotional Engagement Enhanced and Predicts Joint Attention and Cooperation in Young Chimpanzees [file desc0017-0682-sd1.docx]

Supplementary Information: **Emotional Engagement Enhanced and Predicts Joint Attention and Cooperation in Young Chimpanzees**

**Supplemental Material and Methods**

## Calculation of Caregiver: Infant Ratio:

*Standard Care (ST) Nursery Staff :* There were 3 staff assigned to the GA Nursery, each worked 5 days/week. From 9am -5pm, there were 2 caretakers on 6 days and 3 caretakers on 1 day; note than from 5pm - 9am there was only 1 caretaker, who was responsible for all the great ape wing in addition to the nursery. In the years of 1987-1990, there were 5, 6, 7, 9 chimpanzee infants respectively that were placed in the nursery. In 1991, 7 infants entered the nursery (5 were given RC); in 1992, 7 infants entered the nursery (4 were given RC); in 1993, 4 infants entered the nursery (all given RC), and in 1994, 5 infants entered the nursery (all given RC). On average, chimpanzees stayed in the nursery for 4 years before moving out to larger enclosures in the Great Ape Wing, where most became integrated into mixed age social groups. The calculation for the caregiver : infant ratio for ST was (2 staff for 6 days, 3 staff on 1 day) 2.14 staff for (27+29+30+27+23 total number of chimpanzees in nursery each calendar year)/5years= 2.14 staff for 27.2 chimpanzees, or 1 caregiver to 12.7 chimpanzees. On average, 1 ST staff provided care for 12-13 chimpanzees.

*Responsive Care (RC) Nursery Staff and Research Staff:* In the research-based responsive care program (RC), for 4 hours per day the caregiver : infant ratio was much reduced. The RC research staff provided all care for the chimpanzees each afternoon during the week. There were at least 4 staff for the 17 RC infants ((5+4+4+5 total number of chimpanzees in each calendar year across the 4 years = average of 4.5 chimpanzees per year, therefore 4staff :4.5 RC chimpanzees, or a caregiver: infant ration of 1:1.25. On average, 1 RC staff provided care for 1-2 chimpanzees.

## Calculation of Caregiver daily contact time:

*Standard Care (ST) caregiver contact:* In the ST laboratory nursery, each chimpanzee from 3-12 months, was fed a bottle and their diapers were changed every 4 hours. Solid food was introduced during the latter half of the chimpanzees’ first year. These caretaking activities lasted approximately 15-20 min. ST staff also moved the infants out of their night cages to their day rooms and each infant was weighed daily, which took approximately 5 min. At the end of the day, each chimpanzee was returned to their night cage. Veterinary checks occurred for less than 5 min daily when infants were between 3 and 5 months, and as needed thereafter. Therefore, in a typical 8-hour 'day' each chimpanzee infant in the ST nursery would experience a maximum total of 60 minutes of hands-on contact with a caregiver (20min for bottle & diaper change twice a day, 5min to move twice a day, 5min check-up = 40+10+5). In the16-hour ‘night’ time, each ST chimpanzee infant in the Great Ape Nursery would experience approximately 80 minutes of contact with a caregiver, i.e., 15-20 min every 4 hours for bottle & diaper changing. Note that chimpanzee infants in the Yerkes nursery remained in continuous contact with the chimpanzees in their peer group, 24hr/7day.

*Responsive Care (RC) caregiver contact:* Each afternoon, Monday through Friday, the RC chimpanzees were fed on demand, experienced considerable movement around the facilities every hour, and were in constant contact with research staff (100% of 4 hours each weekday afternoon time =240min + 60 morning contact as ST chimpanzee computed above). Note that this is 4 hours per day for 5 days a week & for the remaining 20 hours of every day, and over weekends, the ratio and caregiver contact time was equivalent to that experienced by ST chimpanzee infants. As in ST, the RC chimpanzee infants were continuously (24 hours, 7 days a week) with their peer groups of 3 to 5 other chimpanzee infants.

**Reliability of scoring procedures**

Eight Multivariate ANOVAs (an analysis at each age) revealed no significant or systematic differences between VB and KG in the scoring of cooperativeness, fearfulness, and positive emotional tone with the ST infant testing (all *p*s ns). Seven Multivariate ANOVAs revealed no significant or systematic differences between KG and JA in IBR scoring with the RC infants (*p*s ns). A single exception was at 7 months, when the IBR scores of JA, the new examiner, were higher than those of KG. We consider this relatively unproblematic because (1) if an examiner's scores were biased by knowledge that the RC nursery was meant to be enriching, then KG should have been higher, but, in fact, JA gave higher ratings at this month; (2) A single one of 16 ANOVAs might be significant based on chance only. (3) Most importantly, we consider a single month of significant examiner difference as unproblematic because the multilevel trajectory analyses were longitudinal, using data from all eight months, and a difference between examiners in a single month could not, in principle or in practice, unduly influence the overall findings. No examiner differences were found in scoring IBR items in 15 of 16 statistical analyses; in other words, all three examiners were consistent in applying the same standard criteria for scoring chimpanzee behavior on the IBR forms.

An additional set of reliability assessments were made on scoring of the BSID items. Intra-rater (same examiner from live and videotaped tests) and inter-rater reliability (chimpanzee examiner versus human expert examiner) assessments on the pass/fail/omitted scoring of all the items of each test revealed very high agreement of 84% and 94%, respectively. Paired t-tests, comparing computed raw scores and computed Mental Development Index (MDI) scores, revealed no significant differences between the chimpanzee examiner and the human examiner expert, *t*(22) = -1.67, *p*> .10, and *t*(22) = -1.47, *p*>.10, respectively, with significant and very high correlations, *r*(23)=.97, *p*<.001, and *r*(23)=.88, *p*<.001, respectively. Therefore, we conclude that the BSID examiners of the chimpanzee infants were consistent and reliable, and performed to the high standard of BSID examiners of humans infants.

**Data summary and analysis**

***Gaze alternation in joint attention items*:** Infants' gaze to the examiner's face, or gaze alternation between the examiner's face and the object, are considered explicit behavioral markers of social engagement, and of coordinating engagement jointly, respectively (Adamson & Bakeman, 1985; Corkum & Moore, 1995). Here we report on gaze alternation, the more conservative marker that the infant engaged jointly with the social partner and the test object, as a validity check that the selected items were indexing JA skills, An independent coding of gaze alternation was conducted from videotapes of 125 test sessions with chimpanzees across all ages for six JA items (#74, #87, #90, #92, #94, #104), revealed that gaze alternation regularly co-occurred during the administration of the JA items (Fig S7), and that chimpanzees from 5months to 12 months used gaze alternation during JA items, i.e., there was no change with age (Fig. S8).. Intra-observer reliability was excellent (kappa = .79, percent agreement 87.5, assessed from 12 test sessions). Therefore we conclude that 'jointness' of the chimpanzee infants activity was explicitly and overtly observable by their gaze alternation during JA items,. Therefore, we are confident that the JA items are valid measures of the skill, as they included both the coordination of attention (demonstrated by gaze alternation), and the coordination of infant activity with that of the social partner (demonstrated by passing the items: Baron-Cohen, 1995; Striano & Reid, 2009; Tomasello et al., 2005; Wieder & Greenspan, 2003).

**Supplemental Figures**

Results for the multilevel model analyses that constrained slopes indicated that all intercepts for ST and RC groups differed, at least marginally, with probability values given in the figure legends (Figures S1 - S6). Slopes for all eight scales differed significantly from zero, with probability values given in the figure legends. A second model allowed slopes to differ for the two chimpanzee groups, and for the scores graphed here the slope of the regression lines for the ST and the RC chimpanzees did not differ.


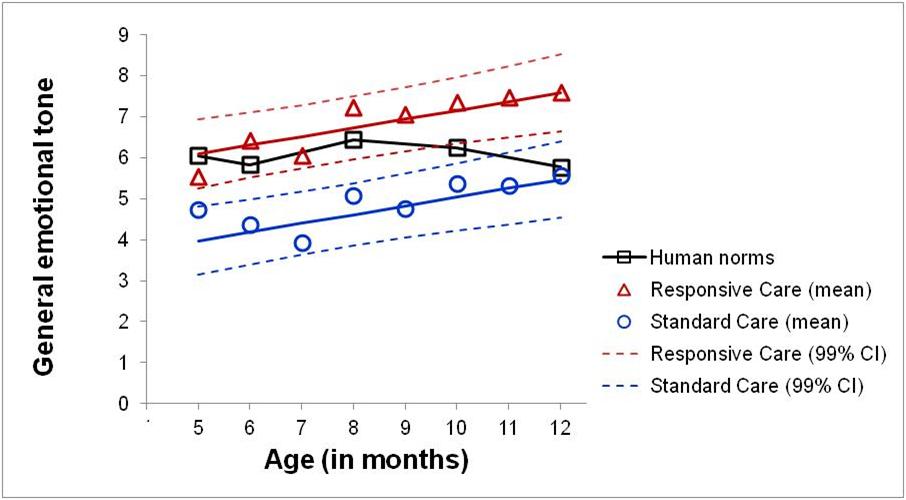


Figure S1: **General Emotional tone**. Positive emotional tone mean scores, developmental trajectory (solid lines) and 99% confidence intervals (CI: dashed lines) are shown for 28 Standard Care (ST) and 17 Responsive Care (RC) laboratory nursery chimpanzees and the 42-57 human infants during Bayley Scales of Infant Development testing. The positive emotional tone of the human infants, at 5mo, 6mo, and 8mo, was not different from that of the RC chimpanzees. At 10mo, the human infants were significantly less positive than the RC chimpanzees but significantly more positive than the ST chimpanzees (*p*s<.01). At 12mo, the humans infants were significantly less positive than the RC chimpanzees, but not different from the ST chimpanzees. The positive emotional tone of RC chimpanzees was significantly higher than ST chimpanzees (*p*<.001). The developmental trajectories of the chimpanzee groups were positive and significant (slope=0.21, *p*<.001) but that of the humans was flat (slope= -0.04).


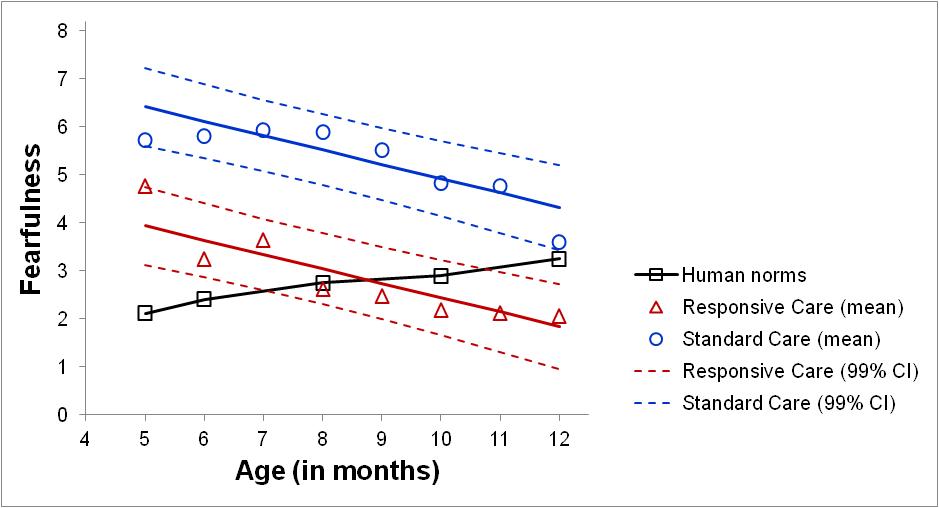


Figure S2: **Fearfulness scores.** Fearfulness mean scores (on a scale from 1=no fear, to 9= continuous fear), developmental trajectory (solid lines) and 99% confidence intervals (CI) are shown for 28 Standard Care (ST) and 17 Responsive Care (RC) laboratory nursery chimpanzees and for 42-57 human infants during Bayley Scales of Infant Development testing. Human infants were significantly less fearful than the RC chimpanzees at 5mo and 6mo (*p*s<.01), but were not different from the RC chimpanzees at 8mo and 10mo. At 12 months, the human infants were significantly more fearful than the RC but significantly less fearful than the ST chimpanzees. RC chimpanzees were significantly less fearful than the ST chimpanzees (*p*<.001). The developmental trajectory for the chimpanzee groups was negative and significant (slope = -0.30, *p*<.001) indicating that there was significant decrease in fearfulness in the chimpanzees, whereas the humans became slightly more fearful from 5 to 12 months (slope=0.16).


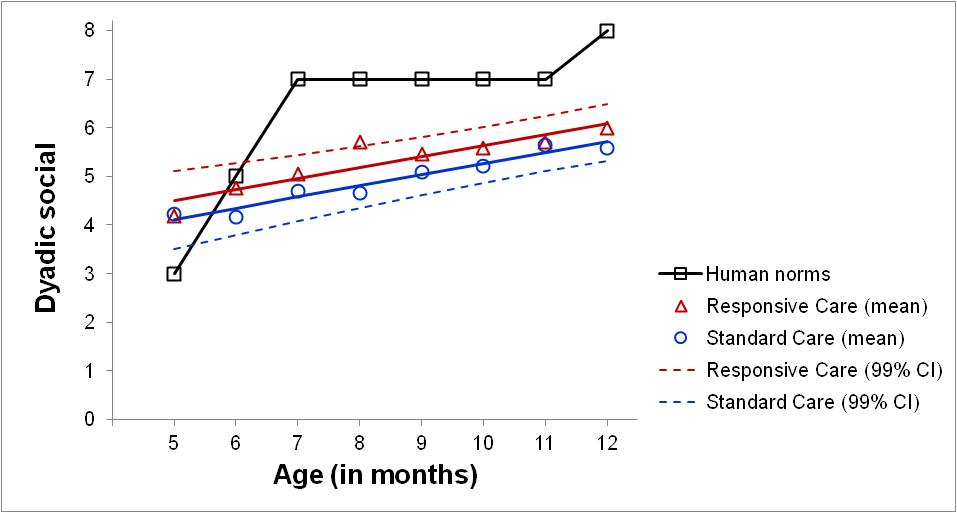


Figure S3: **Dyadic Social Success.** Success in dyadic social items, developmental trajectory (solid lines) and 99% confidence intervals (CI) are shown for 28 Standard Care (ST) and 17 Responsive Care (RC) laboratory nursery chimpanzees and norms for human infants from Bayley Scales of Infant Development testing. The social skill success of human infants was below the chimpanzees at 5mo (*p*<.01), and not different from the RC chimpanzees at 6mo. The laboratory nursery-reared chimpanzees were significantly impaired in social skills compared to these family-raised human infants from 7- through 12-months (*p*s <.01). The social skills of RC chimpanzee were marginally better than the ST chimpanzees (*p*=.08). Developmental trajectories of all groups were positive and significant (*p*s<.001), and the slope of the human infants (*b=*0.71) was higher than those of the chimpanzee groups (*b*s=0.23).


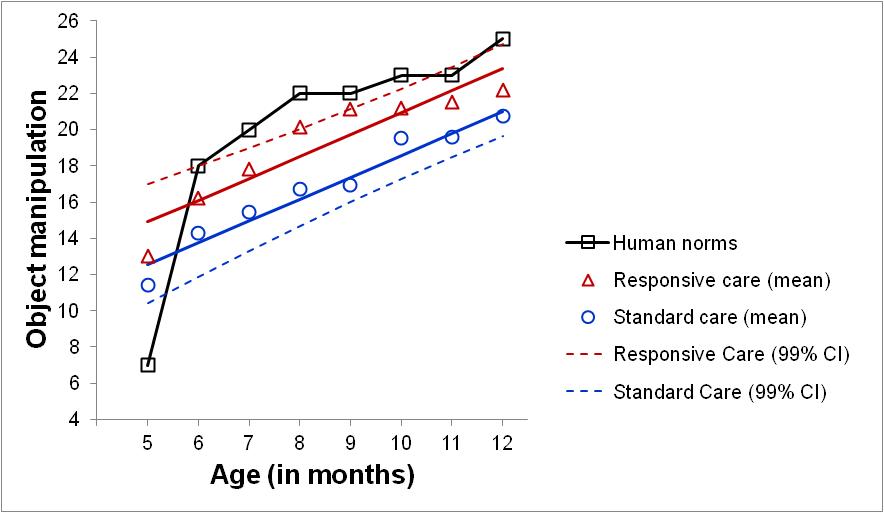


Figure S4: **Dyadic Object Manipulation.** Success in simple manipulation of objects, developmental trajectory (solid lines) and 99% confidence intervals (CI) are shown for 28 Standard Care (ST: blue circles) and 17 Responsive Care (RC: red triangles) laboratory nursery chimpanzees and norms for human infants (black squares) from Bayley Scales of Infant Development testing. Human infants at 5mo were significantly less skillful in object manipulation than the ST chimpanzees (*p*s<.01). Human infants from 6mo through 10mo, and 12mo were slightly higher than the RC chimpanzees (*p*s<.01), but were not different at 11months. RC chimpanzees were significantly more successful than ST chimpanzees (*p*=.002). Developmental trajectories of all groups were positive and significant (*p*s<.001), but the trajectory for human infants (*b* =2.57) was steeper than those of the chimpanzee groups (*b*s=1.21).


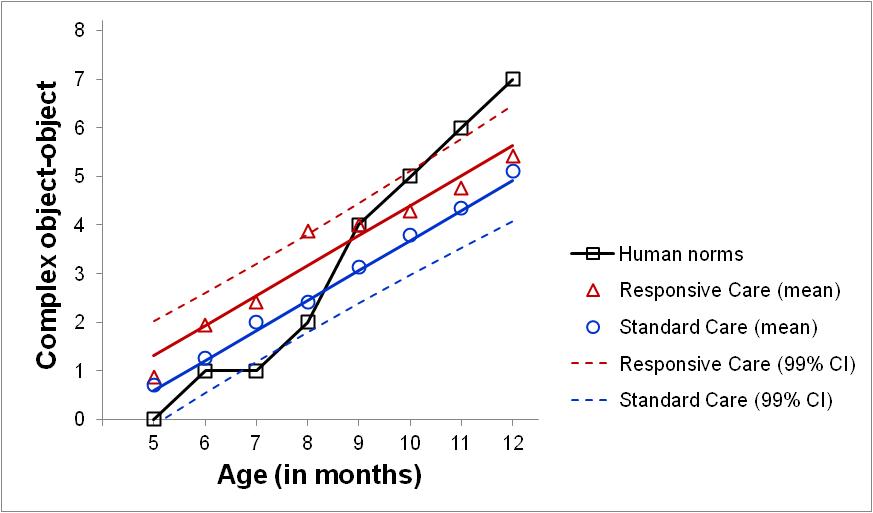


Figure S5: **Triadic Object-Object Manipulations.** Success in triadic object-object manipulation, developmental trajectory (solid lines) and 99% confidence intervals (CI) are shown for 28 Standard Care (ST: blue circles) and 17 Responsive Care (RC: red triangles) laboratory nursery chimpanzees and norms for human infants (black squares) from Bayley Scales of Infant Development testing. The success of human infants in complex object-object manipulation was not different from ST chimpanzees from 5mo through 8mo (*p*s<.01), not different from RC chimpanzees at 9mo and 10mo (*p*s<.01), but was significant higher than RC chimpanzees at 11mo and 12mo (*p*s<.01). The success of RC chimpanzees was marginally higher than that of ST chimpanzees (*p*=.07), with equivalent slopes (*b* =0.62). Developmental trajectories were positive, significant (*p*<.001), and steep for all groups, but steepest for the human infants (*b*=1.00).


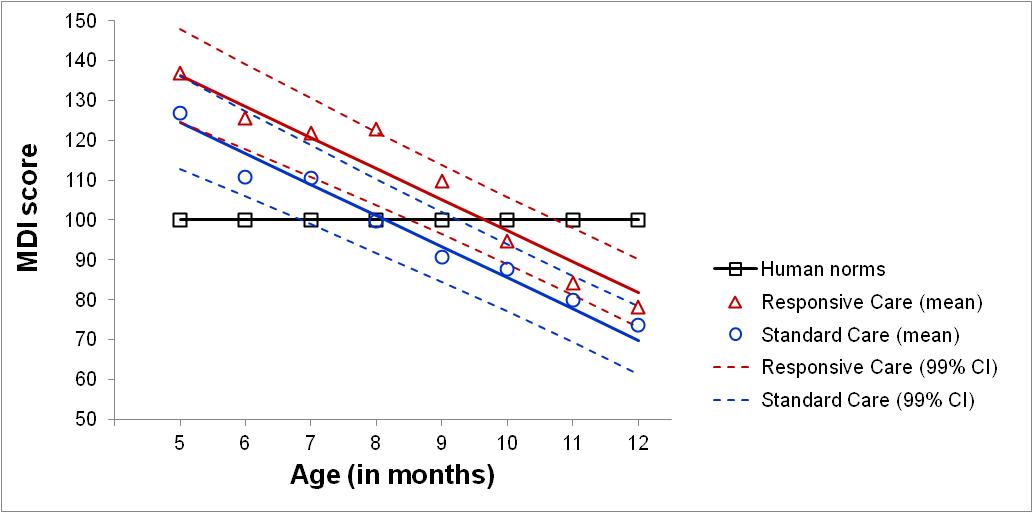


Figure S6: **General Cognition (Mental Developmental Index).** Mental Developmental Index (MDI) mean scores, developmental trajectory (solid lines) and 99% confidence intervals (CI: dashed lines) are shown for 28 Standard Care (ST: blue circles) and 17 Responsive Care (RC: red triangles) laboratory nursery chimpanzees, with normed values for human infants (black squares) from Bayley Scales of Infant Development testing. MDI values of human infants, standardized at 100 (by definition), were significantly below those of the chimpanzees at 5mo and 6mo (all *p*s<.01), not different from the chimpanzees from 7mo through 10mo (*p*s<.01), and significantly the RC chimpanzees from 11mo (*p*s<.01). MDI scores were significantly higher in RC than ST chimpanzees (*p*=.011). MDI developmental trajectories of the chimpanzees were negative, significant (*p*<.001), and steep (*b*= -7.79), in contrast to the 'by-design' flat slope of human norms (Bayley, 1969).


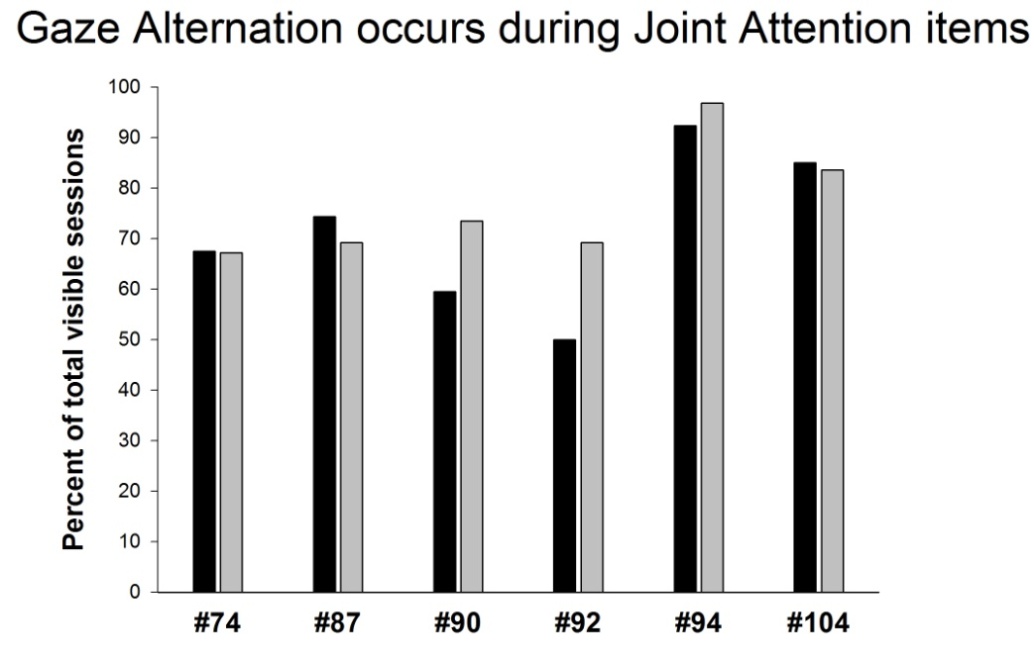


Figure S7: **Chimpanzee Gaze Alternation during Joint Attention items.** Percent of Joint Attention items that included Gaze Alternation, coded from videotapes of 125 test sessions of the Bayley Scales of Infant Development conducted with chimpanzees, from the ages of 5 through 12 months. The columns are the percent of total visible sessions in which the chimpanzee looked at the examiner's face within 3 seconds of looking at the test object, during administration of six JA items (see Table S1 for details of administration and scoring): Item #74 is 'attend to scribbling'; item #87 is 'fingers hole in pegboard' ; item #90 is 'puts cube in cup on command'; item #92 is 'stirs with spoon in imitation'; item #94 is 'inhibits on command'; and item #104 is 'pats whistle doll in imitation'. Black columns are from Standard Care chimpanzees, and grey columns are from Responsive Care chimpanzees. There were no group differences in gaze alternation accompanying joint attention items (chi^2^ values for all six items were ns). For some items (specifically, #92, #94, & #104), gaze alternation occurred significantly more often than 50% of the time, chi^2^(5)=30.06, p<.001.


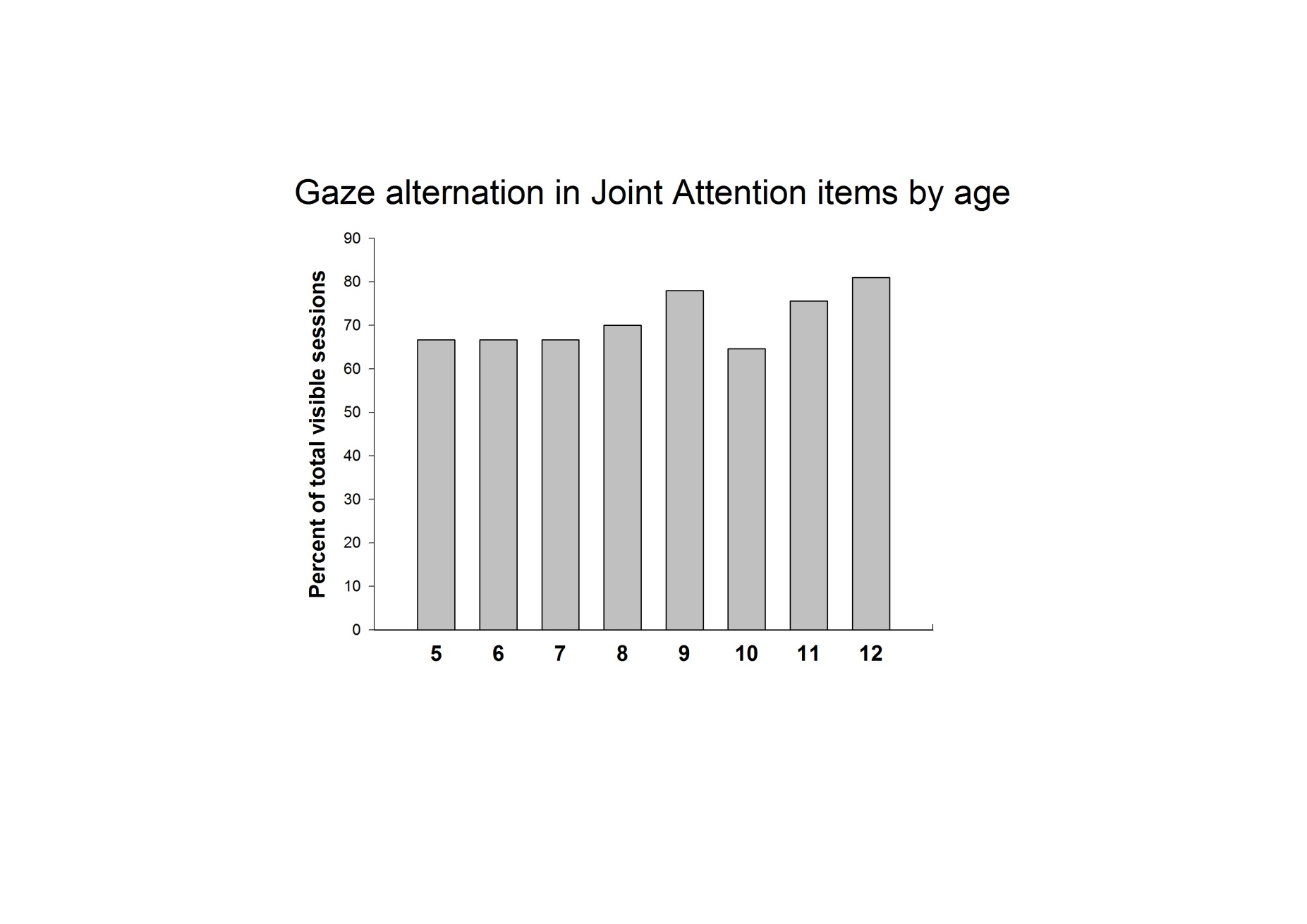


**Age (in months)**

Figure S8: **Chimpanzee Gaze Alternation during Joint Attention by Age**. During 65-80% of monthly sessions, chimpanzee alternated gaze between an object and the face of the examiner during the administration of six Joint Attention items from the Bayley Scales of Infant Development (items are listed in the legend for Figure S7). Each column is the percentage of gaze alternation (GA) for chimpanzees tested on the indicated age. The number of chimpanzees and total number of JA items administered) for which GA was sampled are listed here: 5mo, n=18 (57); 6mo, n=18 (63); 7mo, n=18 (63); 8mo, n=25 (112); 9mo, n=11(51); 10mo, n=17(65); 11mo, n= 9 (41); and 12mo, n=10 (48), respectively. This graph illustrates the lack of age-related changes in gaze alternation during joint attention by chimpanzee infants, from 5 through 12 months of age.

**Supplemental Tables:**

Table S1: **Description of Joint Attention items (abstracted from Bayley, 1969)**. E = Examiner; mo=month

| Test Item # short name (age when 50% of humans pass) | Joint Attention items: Brief Description of Administration and of Scoring |
| --- | --- |
| 74 - Attends to scribbling (5.8mo) | E scribbles with crayon on paper while infant watches. E then directs infant to 'Write'. Pass if infant attends to the demonstrated scribbling. Coordinated agent-object attention. |
| 81 - Cooperates in games (7.6mo) | E initiates a game, such as peek-a-boo with the mirror or pat-a-cake,. This item is passed only if the infant engages in anticipatory behaviors, such as putting hands in pat-a-cake position in advance of the game, or engages actively in searching for hidden E or in revealing E in peek-a-boo. Coordinated agent - object/event attention. |
| 84 - Listens selectively to familiar words (7.9mo) | Either during test or observation, pass if infants shows evidence of listening to words and differential responding (e.g., with facial expression or vocalizations). Sound-meaning reference. |
| 87 - Fingers holes in peg board (8.9mo) | E puts her finger into several holes in the pegboard. Pass if infant pokes a finger in a hole. Imitation of agent's action on object. |
| 89 - Respond to verbal request (9.1mo) | Pass if infant responds, with gesture or word, to any verbal request, such as 'wave bye-bye', or 'where's the light?'. Sound-meaning reference. |
| 90- Puts cube in cup on command (9.4mo) | E demonstrates, then gives cube to infant with continued gestures & commands to 'Put it in the cup'. Pass if infant places cube over or into cup in response to command. Imitation or follow requested action on object. |
| 92 - Stirs with spoon in imitation (9.7mo) | E demonstrates, then lays spoon aside the cup. Pass if infant touches spoon to rim or inside of cup, or attempts stirring motion with spoon inside the cup. Imitation of action with object on another object. |
| 94 - Inhibit on command (10.1mo) | Typically when infant puts crayon in mouth, E commands 'No!' Pass if infant inhibits, even momentarily. Follow sound-meaning reference. |
| 95- Attempts to imitate scribble (10.4mo) | E scribbles with crayon on paper while infant watches. E then directs infant to 'Write'. (Same as item 74). Pass if brings crayon to paper. Imitation of action with object on another object. |
| 97 - Repeats performance laughed at (10.8mo) | E laughs appreciatively at some activity of the infant. Pass if infant repeats performance in response. Coordination of infant action with E's reaction to activity. |
| 99 - Pushes car along (11.3mo) | E demonstrates by pushing car across table. E indicates by gesture and word that infant should push it. Pass if infant pushes car with its wheels on the table. Imitation or follow requested action on object. |
| 100 - Puts 3 or more cubes in cup (11.8mo) | E encourages, by gesture or repeated requests, putting more cubes in cup after infant has put one in (item #90). Pass if 3+cubes are in the cup. Follow requested action with object. |
| 104 - Pats whistle doll, in imitation (12.2mo) | E hits doll smartly to produce whistle a few times then encourages infant to do the same. Pass if infant imitates the hitting motion on the doll. Imitation or follow requested action on object. |
| 105 - Dangles ring by string (12.4mo) | E dangles the ring by the string before laying it down on table. Pass if, after securing ring, infant dangles it in imitation. Imitation of agent's action on object. |
| 111- Builds tower of 2 cubes (13.8mo) | E puts several cubes in front of infant, stacks 3 while saying, "Let's build a house. See?" By word and gesture, E asks infant to make a house. Pass if within 3 trials, infant balances a cube on top of another. |
| 114 - Puts 9 cubes in cup (14.3mo) | E encourages, by gesture or repeated requests, putting more cubes in cup (after item #90 & item # 100). Pass if all 9 cubes are inside the cup. |
| 115 - Closes round box (14.6mo) | E demonstrates opening and closing round box several times, and then verbally requests that infant put the cover on. Pass if infant succeeds in closing the box. |
| 116 - Uses gestures to make wants known (14.6mo) | E observes infants efforts to communicate. Pass if infant makes gesture to ask for, or to refuse toys or other things. |
| 117 - Shows shoes or other clothing, or own toy (15.3mo) | E asks "Where are your shoes?" or "Show me your shoes" or some other article of clothing or a toy that the infant knows by name. Pass if, in response to word, infant points to, touches, or looks at the named item. |
| 122-Attains toy with stick (17.0mo) | E demonstrates pulling toy bunny to infant using the stick, say '[name] make the bunny come!'. Pass if infant makes purposeful attempt, even if does not secure toy. |
| 128-Point to parts of doll (19.1mo) | Request infant to show, or point to, parts of the doll (record each shown, hair, mouth, ears, hands, eyes, feet, & nose). Pass if infant points out at least 3 parts. |

Table S2: **Metrics of Joint Attention items**: percent of chimpanzees passing at each age, and the age range (from when 3% pass to when 100% pass) for humans from the Bayley manual (1969), and computed here for chimpanzees. Mean age, when given, is the age at which 50% of the chimpanzees pass the item. ? indicates unknown, outside of the ages tested.

| **Joint Attention items of the Bayley Scales of Infant Development** | | | |
| --- | --- | --- | --- |
| Test Item # short name | Age range (mo)  **Human** | Age range (mo)  **Chimpanzee Mean** | % of total chimpanzees that pass item at each age |
| 74 - Attends to scribbling | 4 - 10 | <5 - 7 (M=<5) | 89% pass at 5 months, 93% 6mo, 95% 7mo, 100% 8mo, 98% 9mo, 100% 10mo, 92% 11mo, 100% 12mo |
| 81 - Cooperates in games | 5 - 12 | <5 - 12+ (M ~ 6) | 41% pass at 5 months, 53% 6mo, 60% 7mo, 73% 8mo, 63% 9mo, 59% 10mo, 72% 11mo, 79% 12mo, |
| 84 - Listens selectively to familiar words | 5 - 14 | <5 - 12+ (M ~8.5) | 30% pass at 5months, 33% 6mo, 43% 7mo, 41% 8mo, 63% 9mo, 59% 10mo, 78% 11mo, 79% 12mo |
| 87 - Fingers holes in peg board | 6 - 12 | <5 - 12+ (M ~ 9) | 8% pass at 5 months, 8% 6mo, 15% 7mo, 20% 8mo, 73% 9mo, 37% 10mo, 50% 11mo, 47% 12mo |
| 89 - Respond to verbal request | 6 - 14 | 12 - ? (M ?) | 3% pass at 5 months, 0% 6mo, 0% 7mo, 0% 8mo, 3% 9mo, 0% 10mo, 2% 11mo, 6% 12mo |
| 90- Puts cube in cup on command | 6 - 13 | 12 - ? (M ?) | 2% passed at 10mo, 0% 11mo, 6% 12mo |
| 92 - Stirs with spoon in imitation | 8 - 15 | 8 - ? | 5% pass at 8mo, 3% 9mo, 2% 10mo, 0% 11mo, 0% 12mo |
| 94 - Inhibit on command | 7 - 17 | <5 - 12+ (M=8) | 11% pass at 5 months, 20% 6mo, 30% 7mo, 51% 8mo, 53% 9mo, 63% 10mo, 70% 11mo, 91% 12mo |
| 95- Attempts to imitate scribble | 7 - 15 | 5 - ? (M?) | 5% pass at 5 months, 3% 6mo, 8% 7mo, 10% 8mo, 3% 9mo, 10% 10mo, 2% 11mo, 6% 12mo |
| 97 - Repeats performance laughed at | 8 - 17 | <5 - 12+ (M ?) | 23% pass at 7mo, 32% 8mo, 18% 9mo, 34% 10mo, 32% 11mo, 44% 12mo |
| 99 - Pushes car along | 8 - 15 | 5 - ? (M ?) | 5% pass at 5 months, 13% 6mo, 15% 7mo, 24% 8mo, 25% 9mo, 37% at 10mo, 22% 11mo, 15% 12mo |
| 100 - Puts 3 or more cubes in cup | 9 - 18 | ? | 3% 12mo |
| 104 - Pats whistle doll, in imitation | 8 - 19 | 5.5 - ? | 3% pass at 5 months, 10% 6mo, 10% 7mo, 22% 8mo, 20% 9mo, 34% 10mo, 30% 11mo, 29% 12mo |
| 105 - Dangles ring by string | 7 - 18 | 5.5 - ? | 3% pass at 5 months, 13% 6mo, 5% 7mo, 15% 8mo, 8% 9mo, 10% 10mo, 15% 11mo, 12% 12mo |
| 111 - Builds tower of 2 cubes | 10 - 19 (M= 13.8) | ? | 3% pass at 5 months, 3% 6mo, 0% 7mo, 0% 8mo, 0% 9mo, 0% 10mo, 0% 11mo, 0% 12mo |
| 114 - Puts 9 cubes in cup | 11 - 20 (M=14.2) | ? | 3% pass at 5 months, 3% 6mo, 0% 7mo, 0% 8mo, 0% 9mo, 0% 10mo, 0% 11mo, 0% 12mo |
| 115 - Closes round box | 10 - 20 (M=14.3) | ? | 3% pass at 5 months, 0% 6mo, 0% 7mo, 0% 8mo, 0% 9mo, 0% 10mo, 0% 11mo, 0% 12mo |
| 116 - Uses Gestures to make wants known | 11-19 (M=14.6) | 7.5 - ? (M ?) | 3% pass at 5 months, 0% 6mo, 3% 7mo, 10% 8mo, 15% 9mo, 17% 10mo, 15% 11mo, 21% 12mo |
| 117 - Shows shoes, clothing, toy | 11-23 (M=15.3) | ? | 3% pass at 5 months, 0% 6mo, 0% 7mo, 0% 8mo, 0% 9mo, 0% 10mo, 0% 11mo, 0% 12mo |
| 122 - Attains toy with stick | 12 - 24 (M=17) | ? | 2% pass at 10mo, 0% 11mo, 0% 12mo |
| 128 - Points to parts of doll | 15 - 26 (M=19.1) | ? | 3% pass at 12mo |

Table S3: **Social Items**. Items selected as Social, with age range for human norms, brief description of administration and scoring criteria (from Bayley, 1969). E=Examiner.

| Social test items from the Bayley Scales of Infant Development | | |
| --- | --- | --- |
| Item # & short title | Human Age range | Description |
| 53 - Mirror image approach | 2 - 7 | E holds mirror and encourages infant to react. Pass if infant approaches with head, body, or hands, or touches the mirror |
| 55 - Vocalizes attitudes | 3 - 8 | Credit if infant vocalizes expressing pleasure, displeasure (but not crying), eagerness, or satisfaction associated with people, toy, or problem activity |
| 58 - Discriminates strangers | 3 - 8 | E notes infant's reaction on arrival or with any other strangers. Pass if infant shows any discriminative behavior to strangers |
| 61 - Likes frolic play | 3 - 8 | E (or M) holds infant, swings, or shakes over head gently. Pass if infant shows pleasure |
| 65 - Smiles at mirror image | 3 - 12 | E holds mirror and encourages infant to react. Pass if infant smiles at mirror image. |
| 76 - Playful response to mirror | 4 - 12 | E holds mirror and encourages infant to react. Pass if infant plays with mirror image, e.g., by patting, banging, reaching, leaning into, or mouthing mirror |
| 79 - Vocalizes 4 different syllables | 5 - 12 | Pass if infant uses at least 4 different well-defined syllables (Note: Chimpanzees did not pass this item) |
| 101 - Jabbers expressively | 9 - 18 | Pass if infant uses any expressive inflections, that are not meaningful words |

Table S4: **Triadic Object-Object Items**: Items selected as Complex Object-Object manipulations with brief description of administration, age range for human norms, and criteria for giving credit (from Bayley, 1969). E= Examiner

| Complex Object-object manipulations | | |
| --- | --- | --- |
| Item# & short title | Human Age Range (months) | Brief description of administration and scoring |
| 71 - Pulls string: secure ring | 4 - 8 | E dangles the ring and then places it on the table with string within reach of the infant. Pass if the infant obtains the ring by own efforts |
| 80 - Pulls string adaptively: secures ring | 5 - 10 | E dangles the ring and then places it on the table with string within reach of the infant. Pass if infant pulls the string in a purposeful manner & obtains the ring or dangles the ring |
| 88 - Picks up cup: secures cube | 6 - 14 | E places desirable small object (e.g., cube) on the table and quickly covers with the cup. Pass if infant lifts cup with goal to pick up hidden object |
| 91 - Looks for contents of box | 8 - 14 | E puts 2 beads in box & rattles, empties the beads on the table & immediately repeat while infant watches. Then outside of infant's view, remove beads and give infant empty blue box. Take back repeat all except second time leave beads in box. Pass if infant looks for beads in box on either first or second trial |
| 96 - Unwraps cube | 8 - 17 | E wraps a desired small toy in paper. Instructs infant to get the toy. Pass if infant unwraps paper in order to find the toy. |
| 102 - Uncovers blue box | 9 - 17 | E puts small toy inside blue box and closes box with lid. Opens lid and removes toy & the hides the toy again, saying "Baby get the toy". Demonstration repeated a few times. Pass if infant removes cover at least twice |
